# A New Genus of Praeaulacidae (Hymenoptera: Evanioidea) from Mid-Cretaceous Kachin Amber: Insights into a Putative New Praeaulacinae Subclade

**DOI:** 10.3390/insects15050351

**Published:** 2024-05-13

**Authors:** Jingtao Yang, Corentin Jouault, Longfeng Li, Chungkun Shih, Dong Ren

**Affiliations:** 1 Institute of Vertebrate Paleontology, College of Life Science and Technology, Gansu Agricultural University, Lanzhou 730070, China; yangjt@gsau.edu.cn; 2 Institut de Systématique, Évolution, Biodiversité (ISYEB), Muséum National d’Histoire Naturelle, CNRS, SU, EPHE-PSL, U A, CP50, 45 Rue Buffon, F-75005 Paris, France; jouaultc0@gmail.com; 3 Institut des Sciences de l’Évolution de Montpellier (UMR 5554), Université de Montpellier, CNRS, F-34095 Montpellier, France; 4 Univ. Rennes, CNRS, Géosciences Rennes, UMR 6118, F-35000 Rennes, France; 5 College of Life Sciences, Capital Normal University, 105 Xisanhuanbeilu, Haidian District, Beijing 100048, China; chungkun.shih@gmail.com (C.S.); 4989@cnu.edu.cn (D.R.); 6 Department of Paleobiology, National Museum of Natural History, Smithsonian Institution, Washington, DC 20013, USA

**Keywords:** Apocrita, new taxon, amber, wing venation

## Abstract

**Simple Summary:**

A new fossil praeaulacid wasp, *Azygdellitha nova* gen. et sp. nov., is described and figured from mid-Cretaceous Kachin amber. This new taxon not only enriches the diversity of praeaulacid wasps from the Kachin amber biota but also provides additional evidence supporting the discussion of a distinct tribe within the subfamily Praeaulacinae.

**Abstract:**

A new praeaulacid genus and species, *Azygdellitha nova* gen. et sp. nov., is described and illustrated based on a male specimen from mid-Cretaceous Kachin amber from Hukawng Valley, Myanmar. This newly discovered taxon increased the diversity of praeaulacid wasps during the Cretaceous period. While this new taxon shares similarities of wing venation with most species of the subfamily Praeaulacinae, it strongly differs from that of three genera previously described from mid-Cretaceous Kachin amber: *Mesevania*, *Paleosyncrasis*, and *Praegastrinus*. We explore the possibility that these genera constitute a distinct tribe within the Praeaulacinae, distinguished by their wing venation. We provide illustrations and emphasize the potentially diagnostic traits supporting this classification.

## 1. Introduction

Members of the superfamily Evanioidea are easily recognized by their metasoma, which is attached in a high position to the propodeum and far from hind coxae. This character is considered an autapomorphy for Evanioidea [1]. Evanioidea comprise three extant families (Aulacidae, Evaniidae, and Gasteruptiidae) and five extinct families (Andreneliidae, Anomopterellidae, Baissidae, Othniodellithidae, and Praeaulacidae) recorded in abundant fossil records [2,3]. 

The family Praeaulacidae encompasses three subfamilies: Cretocleistogastrinae, Praeaulacinae, and Nevaniinae. The Praeaulacidae is the earliest diverged family of Evanioidea and is represented by more than 80 fossil species. Fossil specimens of Praeaulacidae are often documented in compression or imprint fossil deposits but rarely in amber deposits [3,4]. In 2015, the first praeaulacid species found in Kachin amber was described by Li et al. [5], based on one specimen. Subsequently, several amber species have been reported from the same deposit (i.e., Kachin amber), including representatives of the Nevaniinae and Praeaulacinae subfamilies [4,6,7,8]. The Cretocleistogastrinae are documented from Lower Cretaceous deposits of East Asia and Australia [9,10]. The Praeaulacinae are found in the Middle Jurassic deposits of northeastern China and Kazakhstan and in mid-Cretaceous deposits from Kachin [5,10,11,12,13]. Finally, the Nevaniinae are documented from the Middle Jurassic deposits of northeastern China and Kazakhstan and from the mid-Cretaceous deposits of Kachin. The Nevaniinae wasps are unique among evanioid wasps because they possess the first and second petiole-like metasomal segments and are distinctly thinner than the remaining segments [1,6,13]. The decline of Praeaulacidae is a topic of interest but remains poorly understood. There are no documented praeaulacid species postdating the Cretaceous period, suggesting their extinction during the Late Cretaceous. One proposed hypothesis for this decline involves competition with Ichneumonoidea [3]; however, the limited number of Praeaulacidae and Ichneumonoidea species described from mid- and Late Cretaceous deposits impedes the testing of this theory. To address this gap and enhance our understanding of mid-Cretaceous praeaulacid wasp diversity, we describe a new praeaulacine wasp, *Azygdellitha nova* gen. et sp. nov., discovered in mid-Cretaceous Kachin amber.

## 2. Materials and Methods

**Figure 1:**
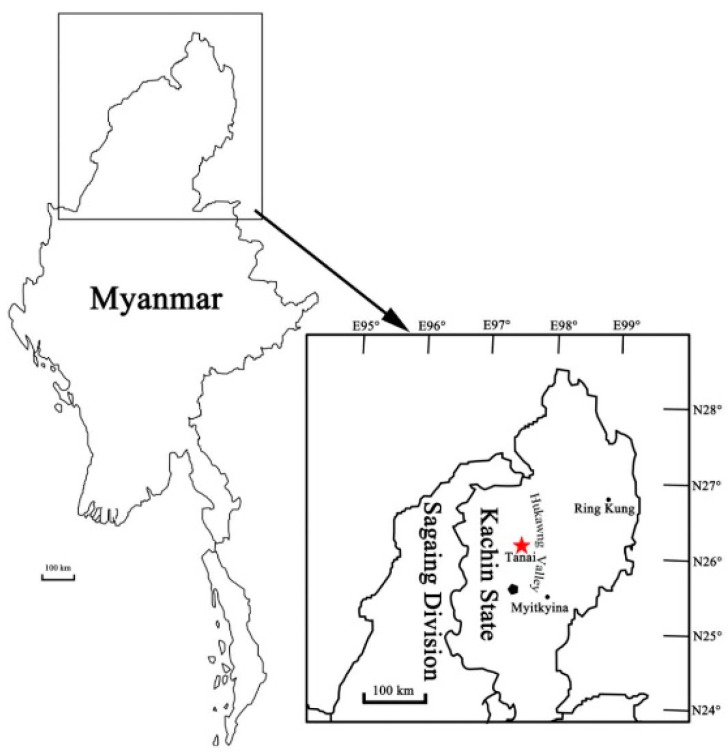
Map of the amber locality near Tanai Village in Hukawng Valley, red star refers to the amber deposit, hexagon refer to Kachin (after [5]).

The amber piece containing the specimen was derived from the deposits of the Hukawng Valley (Kachin, Northern Myanmar). The amber deposits are approximately 100 km southwest of the Village of Tanai (Figure 1). For Kachin amber, the geological age has been estimated at about an early Cenomanian age (98.79 ± 0.62 Mya) based on zircon U-Pb dating [14,15,16]. The mines have yielded abundant insect inclusions, which were later used to illustrate many key evolutionary characters and behaviors (e.g., the early evolution of wing scales in moths and butterflies, and ovipositor sensilla in Hymenoptera) [17,18,19,20,21,22,23]. In recent years, many well-preserved Evanioidea and insect fossils have been reported form this deposit [2,3,4,5,6,24,25,26,27,28].

The type specimen reported herein is housed in the College of Life Science and Technology, Gansu Agricultural University, Lanzhou City, Gansu Province, China. The male amber was examined and photographed using a Nikon SMZ 25 dissecting microscope (Nikon Precision Shanghai Co., Ltd., Shanghai, China) and a Nikon DS-Ri2 digital camera system (Nikon Precision Shanghai Co., Ltd., Shanghai, China). Morphological terminology used here is based on Jouault and Nel [4].

## 3. Results

Systematic palaeontology

Order Hymenoptera Linnaeus, 1758

Suborder Apocrita Gerstaecker, 1867

Superfamily Evanioidea Latreille, 1802

Remarks: The new specimen belongs to Evanioidea based on its metasoma articulated high on the propodeum and well above the metacoxae [29].

Clade Protoevanioides Jouault et al., 2022

Family Praeaulacidae Rasnitsyn, 1972

Remarks: The new specimen is placed within the family Praeaulacidae because of its complete fore wing venation (with two rs-m and two m-cu crossveins) and its hind wing venation with Cu, rs-m, and cu-a present. Additionally, it possesses a fused metasomal segment 1 (vs. metasomal segment 1 separated in Baissidae); a metasomal segment 1 with the anterior margin distinctly separated from the metanotum (vs. metasomal segment 1 with the anterior margin almost touching the metanotum in Gasteruptiidae); a long, cylindrical metasoma (vs. a short, ovate metasoma with distinct petiole in Evaniidae and a broad, oval metasoma in Aulacidae); a head without a cephalic horn and hind wings with Cu and cu-a present (vs. a head with a cephalic horn and hind wing venation lacking distinct Cu and cu-a in Othniodellithidae; [26]). The fore wing of the new specimen has a complete 2rs-m crossvein (vs. absent in anomopterellid wasps), which cannot be attributed to the family Anomopterellidae [30]. The new amber differs from Andreneliidae in that it has a comparatively thin and elongated pterostigma [31]. 

The new specimen cannot be placed within the Nevaniinae because of its one-segmented petiole [1,6,32]. It further differs from representatives of the latter subfamily because of its fore wing with a vein M + Cu longer than the combined lengths of the veins 1-Rs and 1-M [1]. We also ruled out affinities of the new specimen with the Cretocleistogastrinae because of its fore wing venation with a thin pterostigma (vs. wide in Cretocleistogastrinae), a 3rs-m crossvein that is completely preserved (vs. completely lost), and a short abscissa between Rs + M and 2-M (vs. absent) [9,33]. Therefore, we place the new fossil within the subfamily Praeaulacinae.

Subfamily Praeaulacinae Rasnitsyn, 1972

*Azygdellitha* Yang, Jouault, Li, Shih, and Ren, gen. nov.

urn:lsid:zoobank.org:act:02296CDB-2055-4319-BB90-8981E8F5E1BF

Etymology: The new generic name is a combination of the Greek words, ‘*azygos*’ (sole, unpaired) and ‘*dellithos*’ (meaning, a kind of “wasp” or “wasp-like”). The gender of the name is feminine.

Diagnosis: Large body size (longer than 9 mm). Head large elongated in length with large eyes, occupying most of head lateral surface; cephalic horn or projection above the antennal bases absent. Antenna with scape more than 3 times as long as pedicel. Fore wing with pterostigma long and thin; 1-M longer than 1-Rs; 1-Rs about as long as its distance to pterostigma, inclined, and reaching Sc+R close to pterostigma; 1cu-a interstitial with M+Cu fork; 2r-rs long and straight, reaching Rs at 2rs-m level, originating from pterostigma apical third; 2rs-m, 3rs-m, 2m-cu present; cell 2rm triangular, longer than wide; cell 3rm rectangular, longer and wider than cell 2rm, with subparallel sides; 2m-cu reaching M distad 2rs-m (i.e., in cell 3rm), closer to 2rs-m than 3rs-m along M. Hind wing with 1-Rs long and nearly straight; distal Rs abscissa fully developed; rs-m long and arched, longer than cu-a; 1-M longer than 1-Rs; cu-a straight, about 1/3 length of 1-M; distal M and Cu abscissae fully developed. Mesosoma narrower than head, with propodeum about 3 times as long as metanotum; tibial spurs formula 2-2-1. Petiole widening after mid-length (slightly pear-shaped), longer than the second gaster segment; gaster long cylindric.

Composition: Type species only. 

Remarks: Currently, six praeaulacine genera, *Archeogastrinus* Jouault and Rosse-Guillevic, 2023 [8]; *Habraulacus* Li, Rasnitsyn, Shih, and Ren, 2015 [5]; *Hadraulacus* Li, Shih, and Ren, 2023 [7]; *Mesevania* Basibuyuk and Rasnitsyn, 2000 [33]; *Paleosyncrasis* Poinar, 2019 [27]; and *Praegastrinus* Jouault and Nel, 2024 [4], are documented from mid-Cretaceous Kachin amber [4,5,7,8,27,34].

**Figure 2:**
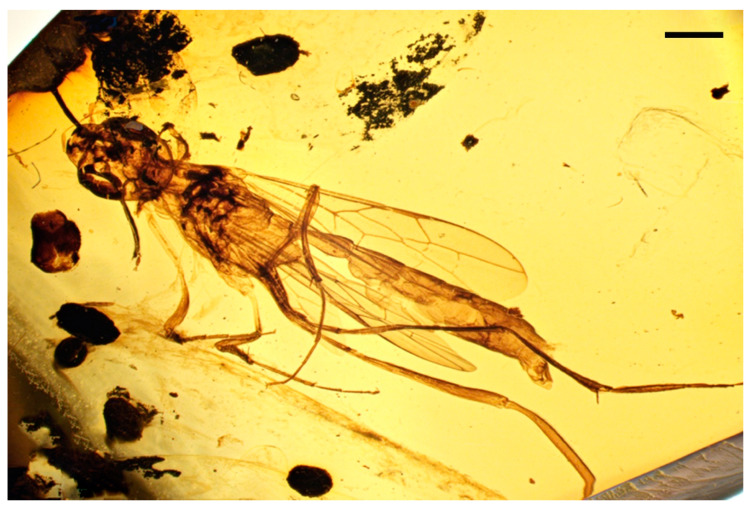
*Azygdellitha nova* gen. et sp. nov. Holotype GAU-HYM-MA-2016008. Habitus, lateral views. Scale bars = 1 mm.

**Figure 3:**
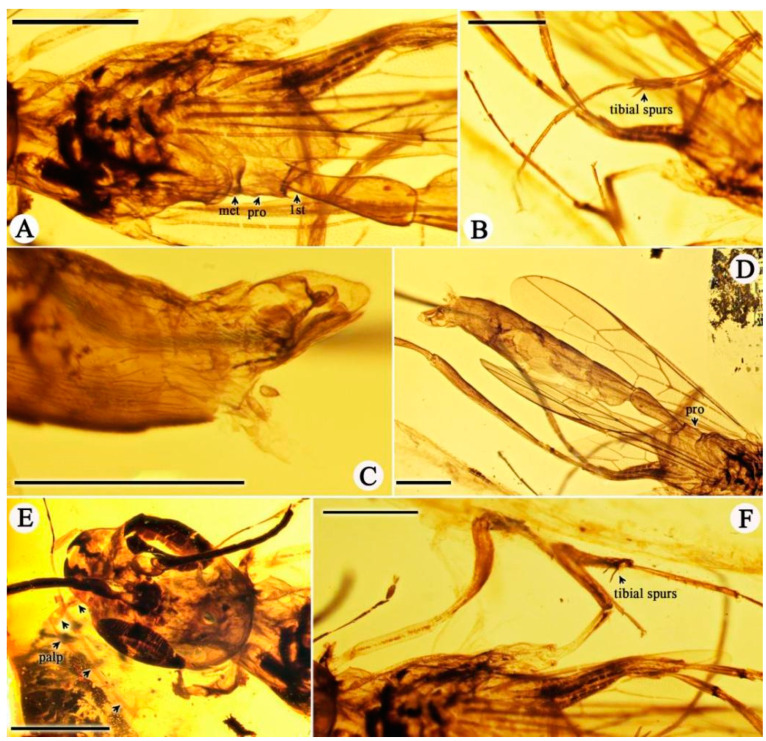
*Azygdellitha nova* gen. et sp. nov. Holotype GAU-HYM-MA-2016008. (**A**) Mesosoma with petiole. (**B**) Mid-leg. (**C**) Male genitalia. (**D**) Metasoma and wings. (**E**) Head. (**F**) Fore and mid legs. met, metanotum; pro, propodeum; 1st, first metasomal segment; palp, palpomeres. Scale bars = 1 mm.

**Figure 4:**
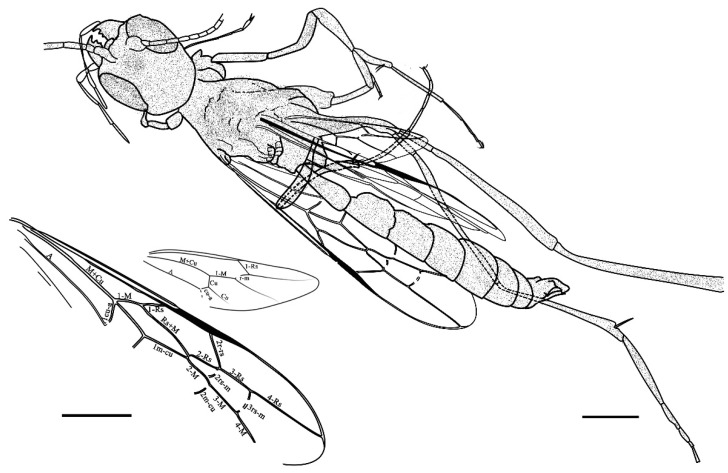
*Azygdellitha nova* gen. et sp. nov. Holotype GAU-HYM-MA-2016008. Line drawing of body and wings with names of veins labelled. Scale bars = 1 mm.

*Azygdellitha nova* Yang, Jouault, Li, Shih, and Ren, gen. et sp. nov. (Figure 2, Figure 3 and Figure 4)

urn:lsid:zoobank.org:act:B77A02D2-B3D3-4D11-B8F4-E3AFAE99CFD1

Type material: Holotype, Male, GAU-HYM-MA-2016008, except both of fore and hind wings folded and antenna partly preserved, body nearly well-preserved.

Etymology: The specific name means new and refers to the new species in Kachin amber, which is derived from the Latin adjective *novus*.

Type locality and horizon: Hukawng Village, Kachin State, Northern Myanmar; Mid-Cretaceous Cenomanian period.

Description: Male. Total body length about 9.3 mm long (from mandible to metasomal apex); fore wing length 5.6 mm; integument largely dark brown, wings clear and hyaline, veins brown in preserved color. 

Head massive, elongated, with broadly rounded posterior corners, 1.81 mm long and 1.44 mm wide; compound eye ovoid, occupying more than half length of head, 0.89 mm long and 0.50 mm wide; antenna filiform (1.73 mm as preserved), with scape distinctly longer than pedicel; mandibles almost triangle-shaped, left mandible with four teeth, apical tooth the longest, first and second marginal teeth of similar length and shape, separated by broad v-shaped edges, third marginal tooth the shortest, blunt, right mandible with four teeth, apical tooth the longest, second marginal tooth slightly shorter than first, third minute and blunt; maxillary palpus elongated, with five palpomeres, about 1.64 mm, basal palpomere wider but shorter than remaining palpomeres, combined lengths of third to fifth palpomeres longer than eye length. 

Mesosoma laterally compressed, longer than high, 2.52 mm long and 1.58 mm high; mesosoma with propodeum weakly areolate, about 3 times as long as metanotum. Other parts of mesosoma poorly preserved. Legs slender, nearly completely preserved except for the tarsus of right hind leg. Hind legs distinctly longer than fore and mid legs, tarsi pentamerous; femora lengths (fore femur 0.98 mm, mid femur 1.46 mm, hind femur 2.85 mm); tibiae lengths (fore tibia 1.09 mm, mid tibia 1.66 mm, hind tibia 2.11 mm); tarsi lengths (fore tarsus 1.93 mm, mid tarsus 2.23 mm, hind tarsus 2.92 mm as preserved). Apex of fore and mid tarsi with a arolium, claws bidentate.

Fore wing long, about 6 mm long, 0.10 mm wide, distinctly longer than wide; 1-M (0.37 mm long) slightly curved, aligned with 1-Rs (0.27 mm long); M+Cu fork interstitial with 1cu-a; 2r-rs (0.48 mm long) straight and longer than 1-M; Rs abscissae from 2-Rs to 4-Rs nearly straight and aligned, reaching wing margin before apex; marginal cell elongated, more than three times as long as wide; two rs-m crossveins present, 2rs-m slightly anteriad 2r-rs along Rs, distinctly anteriad 2m-cu along M; 3rs-m (0.35 mm long) bent, distinctly longer than 2rs-m (0.18 mm long); third submarginal cell 1.4 times as long as second submarginal cell; Rs+M about twice as long as 2-Rs; 1m-cu shorter than Rs+M; first discal cell nearly slanted quadrangle with four sides not equal in lengths; 2m-cu present; A slightly longer than M+Cu, meeting 1cu-a. Hind wing about 3.09 mm long, with venation nearly complete, except lacking C; 1-Rs longer than rs-m, but shorter than 1-M; Cu and cu-a present, forming a wide ‘Y’ shaped angle; angle between 1-M and 1-Cu smaller than angle between first and second Cu abscissae; jugal lobe lacking.

Metasoma 1.13 mm long (including petiole); gaster elongated, 4.16 mm long, second segment to fourth segment nearly equal in lengths and widths, fifth segment slightly wider, sixth and seventh gradually shorter in lengths. Male genitalia outside of the gaster, clasper broad and covered by setae.

## 4. Discussion

Comparisons with other Praeaulacidae from the Kachin amber biota

**Figure 5:**
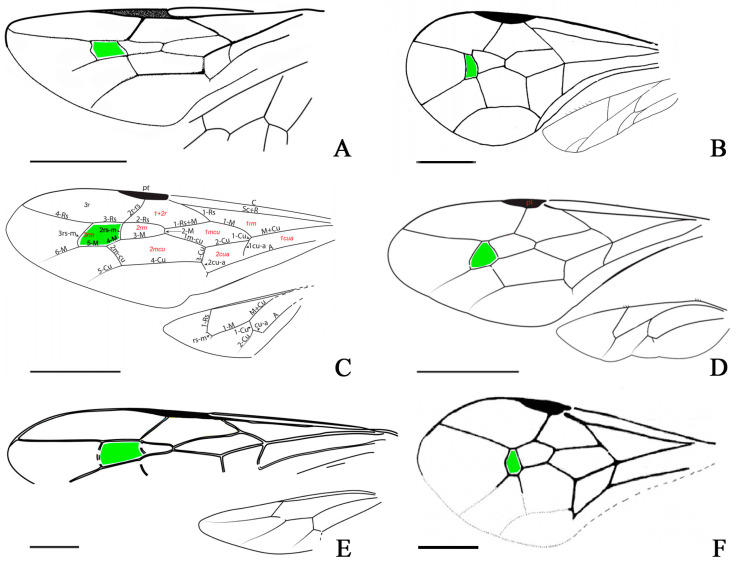
Wings of six species in Praeaulacidae: (**A**) *Habraulacus zhaoi* Li, Rasnitsyn, Shih, and Ren, 2015; (**B**) *Paleosyncrasis hongi* Poinar, 2019; (**C**) *Archeogastrinus kachinensis* Jouault and Rosse-Guillevic, 2023; (**D**) *Praegastrinus edithae* Jouault and Nel, 2019; (**E**) *Azygdellitha nova* Yang, Jouault, Li, Shih, and Ren, gen. et sp. nov.; (**F**) *Mesevania swinhoei* Basibuyuk and Rasnitsyn, 2000. Scale bars = 1 mm. Green color cells refer to 3rm.

As shown in Figure 5, the new species can be easily differentiated from *Archeogastrinus* at least because its head and fore wing are more elongated (vs. rounded or quadrate in *Archeogastrinus*), its fore wing has an interstitial 1cu-a with 1-M (vs. 1cu-a postfurcal), and its hind wing has a long curved r-m (vs. short and straight), and its distal abscissae of Rs, M, and Cu are long (i.e., nearly reaching the wing margin vs. short) [8]. Recently, the genus *Hadraulacus* was described from mid-Cretaceous Kachin amber, and it stands out among the Praeaulacidae of this deposit because it lacks the vein 2rs-m in the fore wing [7]. The wing venation of *Habraulacus* can serve as a good example to differentiate it from the new specimen because it has a long 1-Rs vein (longer than 1-M), while it is shorter in the new specimen; 2r-rs meets Rs well before 2rs-m (vs. being close to 2rs-m in the new species); and 2-M is present and fully developed (vs. much shorter) [5]. Similarly, the hind wing venation of the *Habraulacus* strongly differs from that of the new species: 1-Rs is short (vs. long), and 1-Cu is shorter than cu-a (vs. cu-a longer shorter Cu) [5]. The rest of the fore wing venation being strongly deformed, we refrain from proposing a complete comparison of its venation with that of the new specimen. 

The new specimen cannot be attributed to the genus *Mesevania* because of its elongated body with a tubular metasoma (vs. a short body and a metasoma virtually rounded in *Mesevania*), its fore wing with 1-Rs shorter than 1-M (vs. longer than 1-M), its crossvein 2r-rs meeting Rs close to 2rs-m (vs. well before), and its crossvein 3rs-m located just after the 2rs-m (vs. well after) [26]. Similarly, it cannot be attributed to the genus *Paleosyncrasis* because of its tubular and elongated metasoma (vs. short and rounded in *Paleosyncrasis*); its long and relatively thin fore wing (vs. short and broad), with 2r-rs meeting Rs close to 2rs-m (vs. well before) and its crossvein 3rs-m located well after 2rs-m (vs. just after 2rs-m); and its hind wing without Rs + M (vs. with a long Rs + M vein and a distal reemergence of both Rs an M) [20]. Finally, the new specimen differs from *Praegastrinus* at least because it lacks a frons with a bi-lobed ledge covering each torulus dorsally, and it has a fore wing with 1-Rs shorter than 1-M (vs. longer in *Praegastrinus*), a lack of a distinct abscissa of M between Rs+M and 1m-cu (vs. fully developed), the crossvein 2r-rs nearly interstitial with 2rs-m (vs. meeting Rs well before 2rs-m), the cell 3rm rectangular (vs. pear-shaped); and its hind wing with a well-developed 2-Rs (vs. absent) and a long and arched r-m crossvein (vs. short and vertical) [4].

A hidden phylogenetic signal in the fore wing venation of Praeaulacidae?

Praeaulacidae have a fore wing venation that is more complete than that of any other evanioid lineages, sometimes retaining a 1r-rs crossvein that delineates cells 1r and 2r ([1]: fig 9). Wing venation serves as a valuable character for delineating Hymenoptera clades due to its relatively stable nature (compared to Polyneoptera lineages) and relatively clear polarity. Across hymenopteran lineages, there is a general trend of reduction and simplification from the ancestral condition of complex wing venation, along with an enlargement of the pterostigma and reduction in the number of antennal flagellomeres [5,35,36,37]. Evanioidea are no exception to this trend, with early-diverged lineages (*Protoevanioides sensu* Jouault et al., 2022) [3] retaining the most plesiomorphic wing venation, the highest number of flagellomeres, and often larger body sizes (up to 2 cm), while the more derived *Neoevanioides* typically exhibit less complete wing venation (frequently lacking some rs-m and m-cu crossveins), fewer flagellomeres, and often a smaller size. These characters support the backbone topology of the Evanioidea phylogeny [2,3] and can provide insights into additional relationships within evanioid families.

Within Praeaulacinae, we observed that genera can be grouped into two distinct categories based on their fore wing venation. Specifically, the configuration of the cell enclosed by the crossvein 3rs-m (referred to as 3rm) and the relative proximity between 2rs-m and 3rs-m facilitate a straightforward differentiation of the genera *Mesevania*, *Paleosyncrasis*, and *Praegastrinus* from other members of Praeaulacinae (Figure 5). The only other genus bearing a slight resemblance to these aforementioned genera is *Evanigaster* Rasnitsyn, 1972, although the expression of this characteristic shape is not as pronounced as observed in the previously mentioned genera. Notably, *Mesevania*, *Paleosyncrasis*, and *Praegastrinus* are all documented from mid-Cretaceous Kachin amber. Consequently, this distinct wing venation pattern could either be interpreted as an apomorphy, justifying their classification into a new tribe within Praeaulacinae, or as an adaptation to a specific flight type or behavior. Given our current state of knowledge and the limited data for praeaulacid wasp diversity from Kachin amber, we refrain from proposing formal taxonomic revisions. However, with the continuous discovery of new species within the family Praeaulacidae in this deposit, we anticipate being able to test this hypothesis within a phylogenetic framework in the near future.

## 5. Conclusions

Based on a single well-preserved specimen of Praeaulacidae found in mid-Cretaceous Kachin amber, we described a new genus and species, *Azygdellitha nova* gen. et sp. nov. This new taxon not only enriches the diversity of praeaulacid wasps from the Kachin amber biota but also provides additional evidence supporting a putative distinct tribe within the subfamily Praeaulacinae. This new clade would be putatively supported by wing venation characters, notably the shape of the cell 3rm and the placement of the crossvein 3rs-m. Three genera placed in the Praeaulacinae share these wing venation patterns, which may suggest that they are closely related, potentially forming a unique clade exclusive to the Kachin amber biota.

## Data Availability

All data from this study are available in this paper and the associated papers.
